# Beneficial effect of transversus thoracic plane blocks on intensive care unit (ICU) length of stay following cardiac surgery: a trial sequential analysis

**DOI:** 10.1097/JS9.0000000000001095

**Published:** 2024-01-18

**Authors:** Kuo-Chuan Hung, I-Wen Chen, Cheuk-Kwan Sun

**Affiliations:** a Department of Anesthesiology, Chi Mei Medical Center, Tainan City; b Department of Anesthesiology, Chi Mei Medical Center, Liouying, Tainan City; c Department of Emergency Medicine, E-Da Dachang Hospital; d School of Medicine for International Students, College of Medicine, I-Shou University, Kaohsiung City, Taiwan

*Dear Editor*,

We read with interest the systematic review and meta-analysis by Xue *et al*.^1^ evaluating the efficacy of transversus thoracic muscle plane block (TTMPB) for postoperative pain management in cardiac surgery patients. The authors found that, compared to the control, TTMPB could reduce pain scores, opioid consumption, intensive care unit (ICU) length of stay, and postoperative nausea and vomiting^1^. Shortening ICU stay is clinically meaningful, as it indicates faster recovery and may potentially improve long-term clinical outcomes. In fact, a large-scale retrospective study comprising 34 696 patients revealed a positive association between the length of ICU stay and one-year mortality rate regardless of the need for mechanical ventilation^2^. Albeit promising, the findings of that study^1^ warrant further evidence for validation.

Although nine randomized controlled trials were included, the relatively small sample size of 454 patients in that study^1^ raises some uncertainty about the precision of the effect estimates. Trial sequential analysis (TSA) is a statistical technique that combines information size calculations and cumulative meta-analysis to help determine when sufficient evidence has been reached to draw firm conclusions about a treatment effect^3,4^. In a cumulative meta-analysis, studies are added one at a time in order of publication date to assess the impact of each trial on the overall effect size^5^. TSA extends this approach by calculating the required information size, which is the minimum number of patients needed for generating reliable evidence. This required sample size depends on factors like the estimated treatment effect, *α* level, power, and variance of the effect size. The TSA then displays boundaries for defining statistical significance, called trial sequential monitoring boundaries, on the cumulative meta-analysis plot^5^. These boundaries consider the accrued sample size and required information size. If the cumulative *z*-curve crosses the monitoring boundaries before reaching the required information size, firm evidence may have been established earlier than expected^5^. On the other hand, if the *z*-curve does not cross the boundaries, additional evidence is likely still needed. By incorporating trial sequential monitoring boundaries, TSA allows for a more robust evaluation of the evidence in cumulative meta-analyses. Given these advantages, TSA represents an improvement over the traditional meta-analytic approach.

Because the authors did not perform TSA to confirm their findings, we conducted this analysis using the raw data from their meta-analysis^1^ to evaluate the cumulative evidence regarding their result on the length of ICU stay. In brief, we used TSA (TSA version 0.9.5.10 Beta) software to carry out the analysis. We set the type I error (*α*) at 0.05 and power at 0.8. For the intervention effect estimate, we used the mean difference in ICU length of stay of −13.27 h from the original meta-analysis data^1^. For the TSA monitoring boundaries, we used an *α* spending function based on the O’Brien–Fleming approach. As shown in Figure 1, our finding of a crossing of the *z*-curve over the required information size boundary suggests adequate evidence to reach firm conclusions. By effectively managing pain through regional anesthesia techniques such as TTMPB, patients may experience fewer pain-related complications, reduced systemic opioid consumption, and subsequently quicker recovery trajectories. This can translate into enhanced recovery and ICU discharge readiness, potentially decreasing ICU congestion and lowering the healthcare costs associated with prolonged ICU stays. Considering the opioid-sparing effect of TTMPB, further research should investigate its long-term impact on patient satisfaction, rehabilitation progression, and the incidence of chronic postoperative pain. Despite this encouraging finding, TSA has several key limitations. A primary concern is the potential for misuse or misinterpretation if the analysis is designed after data collection, emphasizing the need for pre-specifying criteria, such as anticipated intervention effects and heterogeneity^3^. Another challenge is the fluctuating nature of the required information size and number of trials, which can change with additional trials, making them a ‘moving target’^3^. Additionally, TSA’s heavy reliance on statistical significance and *P* values instead of confidence intervals is problematic, especially when the information size is insufficient, leading to misleading unadjusted confidence intervals^3^.

**Figure 1:**
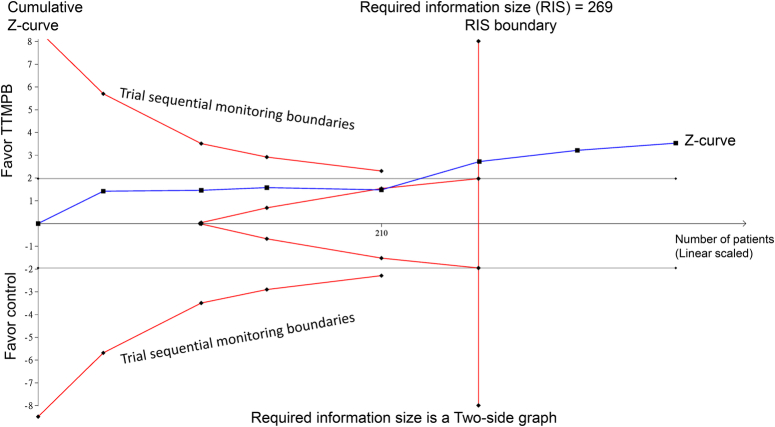
Trial sequential analysis evaluating cumulative evidence about the effect of transversus thoracic muscle plane block (TTMPB) versus control on the length of intensive care unit (ICU) stay. The *z*-curve (blue line) crosses the required information size boundary, suggesting sufficient evidence to draw firm conclusions about the magnitude of reduction in ICU stay with TTMPB.

In summary, while the small overall sample size of the study by Xue *et al*. left some room for uncertainty, our analysis confirmed that TTMPB is beneficial to the shortening of ICU stay as reported in that study, thereby supporting the use of the anesthetic technique in clinical practice.

## Ethical approval

Not applicable.

## Consent

Not applicable.

## Sources of funding

No external funding was received for this study.

## Author contribution

K.-C.H. and C.-K.S.: wrote the main manuscript text; I-W.C.: prepared Figure 1. All authors read and approved the final version of the manuscript.

## Conflicts of interest disclosure

The authors declare no conflicts of interest.

## Research registration unique identifying number (UIN)

Not applicable.

## Guarantor

Kuo-Chuan Hung, I-Wen Chen, and Cheuk-Kwan Sun.

## Data availability statement

The datasets used and/or analyzed in the current study are available from the corresponding author upon reasonable request.

## Provenance and peer review

This paper was not invited.
